# Effects of Chalcogen Atoms on Excited-State Double-Proton Transfer Behavior for 3,6-bis(4,5-Dihydroxyoxazo-2-yl)benzene-1,2-diol Derivatives: A Computational Investigation

**DOI:** 10.3390/molecules29020461

**Published:** 2024-01-17

**Authors:** Dapeng Yang, Chang Liu, Meiyi Zhang, Jinfeng Zhao

**Affiliations:** 1 College of Electronics and Engineering, North China University of Water Resources and Electric Power, Zhengzhou 450046, China; yangdapeng@ncwu.edu.cn; 2 College of Physical Science and Technology, Shenyang Normal University, Shenyang 110034, China; lc20021210202307@163.com (C.L.); meiyizhang12138@163.com (M.Z.); 3 Molecular Sciences and Engineering, Institute of Frontier and Interdisciplinary Science, Shandong University, Qingdao 266237, China

**Keywords:** hydrogen bonding interaction, chalcogen atomic electronegativity, charge transfer, potential energy surface, excited-state double-proton transfer

## Abstract

The impact of the chalcogen atomic electronegativity (O, S, and Se atoms) of new organic molecules on excited-state dynamical reactions is self-evident. Inspired by this kind of distinguished photochemical characteristic, in this work, we performed a computational investigation of chalcogen-substituted 3,6-bis(4,5-dihydroxyoxazo-2-yl)benzene-1,2-diol (BDYBD) derivatives (i.e., BDYBD-O, BDYBD-S, and BDYBD-Se). In this paper, we pay close attention to characteristic BDYBD derivatives that contain intramolecular double hydrogen bonds (O1–H2···N3 and O4–H5···N6). The main goal of this study was to explore how changes in atomic electronegativity affect the way hydrogen bonds interact and how excited molecules affect transfer protons. We go into further detail in the main text of the paper. By fixing our attention to geometrical variations and infrared (IR) vibrational spectra between the S_0_ and S_1_ states, exploring hydrogen bonding behaviors using the core-valence bifurcation (CVB) index, and simulating hydrogen bonding energy (E_HB_) via the atom in molecule (AIM) method, we clarified the photo-induced strengthened dual hydrogen bonding interactions that facilitate the excited-state dual-proton transfer (ESDPT) behavior of BDYBD derivatives. The reorganization of charge stemming from photoexcitation further verifies the tendencies of ESDPT reactions. We relied on constructing potential energy surfaces (PESs) by adopting a restrictive optimization approach, and herein, we finally clarify the gradual ESDPT mechanism of BDYBD derivatives. Particularly, we confirm that the variation in chalcogen atomic electronegativity has a regulatory effect on the ESDPT behavior of BDYBD derivatives; that is, the lower the atomic electronegativity, the more favorable it is for the gradual ESDPT reaction.

## 1. Introduction

The phenomenon of hydrogen bonding has long been acknowledged for its paramount significance across the realms of physics, chemistry, and biology [1,2,3]. The intricate complexity exhibited by hydrogen bonds in solutions has captivated scholars from diverse fields who have tirelessly delved into this subject matter, employing an array of experimental and theoretical methodologies owing to the pivotal involvement of solute–solvent interactions in molecular nonequilibrium processes within liquids [4,5,6]. Despite decades of extensive research shedding light on the electronic ground-state characteristics of hydrogen bonds, our understanding of their behavior in electronically excited states remains somewhat limited. Consequently, unraveling the dynamic response exhibited by intramolecular and intermolecular hydrogen bonds towards photo-induced alterations in charge distribution across different electronic states—commonly referred to as hydrogen bonding dynamics—holds immense interest within the realm of photochemistry.

The phenomenon of proton transfer (PT), which plays a pivotal role in photochemical reactions, is frequently witnessed in nature as an elemental occurrence transpiring along pre-existing hydrogen bonding pathways [7,8,9,10,11,12]. Ever since Weller’s revolutionary discovery back in 1955 [13], investigations into the intriguing characteristics of excited-state intramolecular proton transfer (ESIPT) linked to charge redistribution within analogous compounds have emerged as captivating domains, significantly impacting areas including photophysics, photochemistry, biochemistry, and allied disciplines. It cannot be denied that ESIPT reactional behaviors are involved in our daily lives and our natural world, and they play vital roles that have been reported theoretically and experimentally. Renowned for its exceptional speed, the ESIPT reaction exhibits distinct phenomena and a remarkable Stokes shift resulting from the formation of tautomers in the excited state [14,15,16,17,18]. Organic molecules with ESIPT characteristics have been widely adopted in many fields, like fluorescence probing, laser materials, optical storage, and even photoluminescence. Over time, an increasing number of researchers have dedicated their unwavering attention to demystifying the fascinating ESIPT properties exhibited by these molecules. The ESIPT process relies on the formation of hydrogen bonds between protonic acid groups and neighboring basic sites, which are distinctive characteristics of molecules exhibiting the remarkable ESIPT properties. Depending on photoexcitation, the acidity of the proton donor group becomes stronger, which makes the acceptor groups more basic. These two factors make it easier for isomers to form through proton transfer within or between molecules [19,20,21,22].

It is undeniable that after nearly half a century of relentless exploration and due to the rapid advancement of theoretical and experimental techniques, mankind has substantially elucidated the reaction mechanism governing the single-proton transfer behavior in excited states along a hydrogen bond chain. However, it must be acknowledged that our understanding of the dynamic behavior exhibited by novel molecular systems containing multiple hydrogen bond chains remains in its nascent stage. Among these systems, those encompassing dual-proton transfers have garnered particular attention due to their status as the simplest and most fundamental system class; they serve as an invaluable foundation for delving into scenarios involving multiple hydrogen bond sites [23,24,25,26]. The research in the existing literature focuses on compounds with symmetrical or asymmetrical structures that serve as sites for proton transfer. Understanding the reaction mechanism is crucial to gaining a deeper understanding of the photophysical characteristics of these excited-state compounds. For instance, Song and colleagues used density functional theory (DFT) and time-dependent DFT (TDDFT) methods to elucidate the dynamics of multiple proton-associated excited states in the classical porphycene fluorophore in a solvent phase [27]. Peng et al. strategically designed and synthesized the classical 1,8-dihydroxy-2-naphthaldehyde (DHNA) compound, which was revealed to have a role in the stepwise ESDPT relay reaction by photoexcitation [28]. In short, the theoretical and experimental investigations of ESDPT behavior associated with double hydrogen bonding chains have been recognized as the most basic form of multiple proton transfer behaviors.

**Figure 1:**
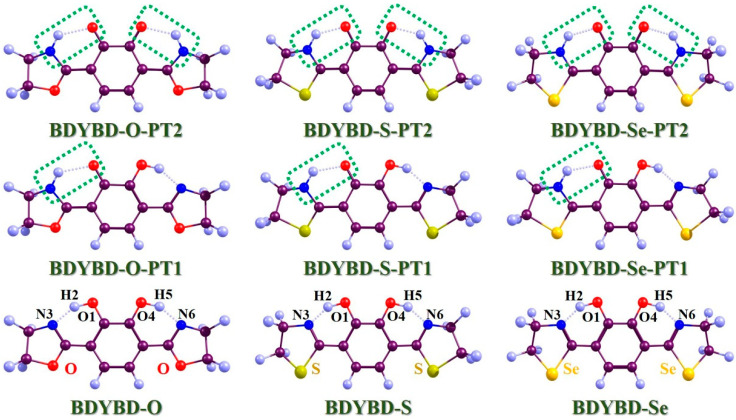
Optimized geometries of BDYBD-O, BDYBD-S, and BDYBD-Se fluorophores. Single-proton transfer tautomers (BDYBD-O-PT1, BDYBD-S-PT1, and BDYBD-Se-PT1) and double-proton transfer tautomers (BDYBD-O-PT2, BDYBD-S-PT2, and BDYBD-Se-PT2) are shown. Dark brownish–red: C; blue–gray: H; red: O; blue: N; chartreuse: S; orange–yellow: Se.

As widely acknowledged, the new types of organic molecules substituted by chalcogen elements have been widely focused on in various fields in recent years. The addition of chalcogen element doping can boost the efficiency of epitaxial quantum while also causing a transfer between singlet and triplet states due to the heavy atom effect [29,30,31,32]. Specifically, Meng and colleagues used an experimental approach to suggest replacing oxygen group elements with sulfur in the traditional molecular system of 3-hydroxyflavone (3HF) [33], showing how this could potentially make ESIPT reactions easier in derivatives of 3HF. This swap also causes a big shift towards the red end of the fluorescence light emitted after the ESIPT reaction. Shi et al. carried out an amazing simulation on the antioxidant activities related to 3HF and its oxygen substitutional derivatives [18], which elegantly unveiled the profound impacts resulting from manipulating atomic electronegativity. It can be seen that the change in the electronegativity of oxygen group elements has a profound effect on the excited-state dynamics of molecules. 3,6-bis(4,5-dihydroxyoxazo-2-yl)benzene-1,2-diol (BDYBD), as a classical molecular fluorophore associated with the dual hydrogen bonds often talked about in the field of excited-state dynamics, was first designed and reported by Enchev and coworkers [34]. Related to bis-3,6-(2-benzoxazolyl)-pyrocatechol [35], BDYBD was reported to present potential two-step reactional behaviors in the S_1_ state experimentally and theoretically [34]. Although previous reports have been able to effectively uncover the dynamics of the BDYBD system in excited states, we still know little about how substituents of oxygen group elements affect the ESDPT reaction in systems with intramolecular hydrogen bonds inspired by atomic electronegativity. In addition, though it Is known that the hydrogen bond of the weak interaction usually requires the consideration of the dispersion effect, this point has been ignored in some simulations [34]. Therefore, considering the issues mentioned above, in this work, we are determined to use a more suitable calculation level to thoroughly explore how changes in the atomic electronegativity of oxygen group elements affect the ESDPT reaction process of BDYBD-O, BDYBD-S, and BDYBD-Se fluorophores. The structures of BDYBD derivatives are shown in Figure 1. Specifically, in this paper, we mainly concentrate on how substituents of oxygen family elements affect the ESDPT behaviors of the BDYBD derivatives mentioned above.

## 2. Results and Discussion 

### 2.1. Geometrical Analyses

In Figure 1, the optimized compounds that we mainly investigated (BDYBD-O, BDYBD-S, and BDYBD-Se) are shown. Correspondingly, the possible proton transfer tautomer geometries are also provided. Our simulations were caried out using ethanol as the solvent. Since we mainly focused on the double hydrogen bonding interaction within the target molecule, it was really important to understand how photoexcitation can directly affect ESDPT behavior. Thus, for an easier understanding of the content discussed here after, we have named these two intramolecular hydrogen bonds as O1–H2···N3 and O4–H5···N6, respectively. Considering the strong rigidity of the molecule and the symmetry of the molecular structure, we found that the structural changes on both sides of the molecule were consistent, which also verified the correctness of our calculation results. In Table 1, we present the optimized molecular structural parameters associated with hydrogen bond O1–H2···N3 for BDYBD-O, BDYBD-S, and BDYBD-Se in the S_0_ and S_1_ states. Due to the symmetry of their structures, O1–H2···N3 and O4–H5···N6 own the same numerical values; thus, we only present the geometrical data of O1–H2···N3 in Table 1. In comparison to the S_0_ state, it is evident that the lengths of the dual hydroxy groups and the distances between H2···N3 and H5···N6 are shortened with the increase in bond angles. These findings suggest that the enhancement of excited-state dual hydrogen bonding interactions can be achieved through photoexcitation [36,37,38,39,40].

**Figure 2:**
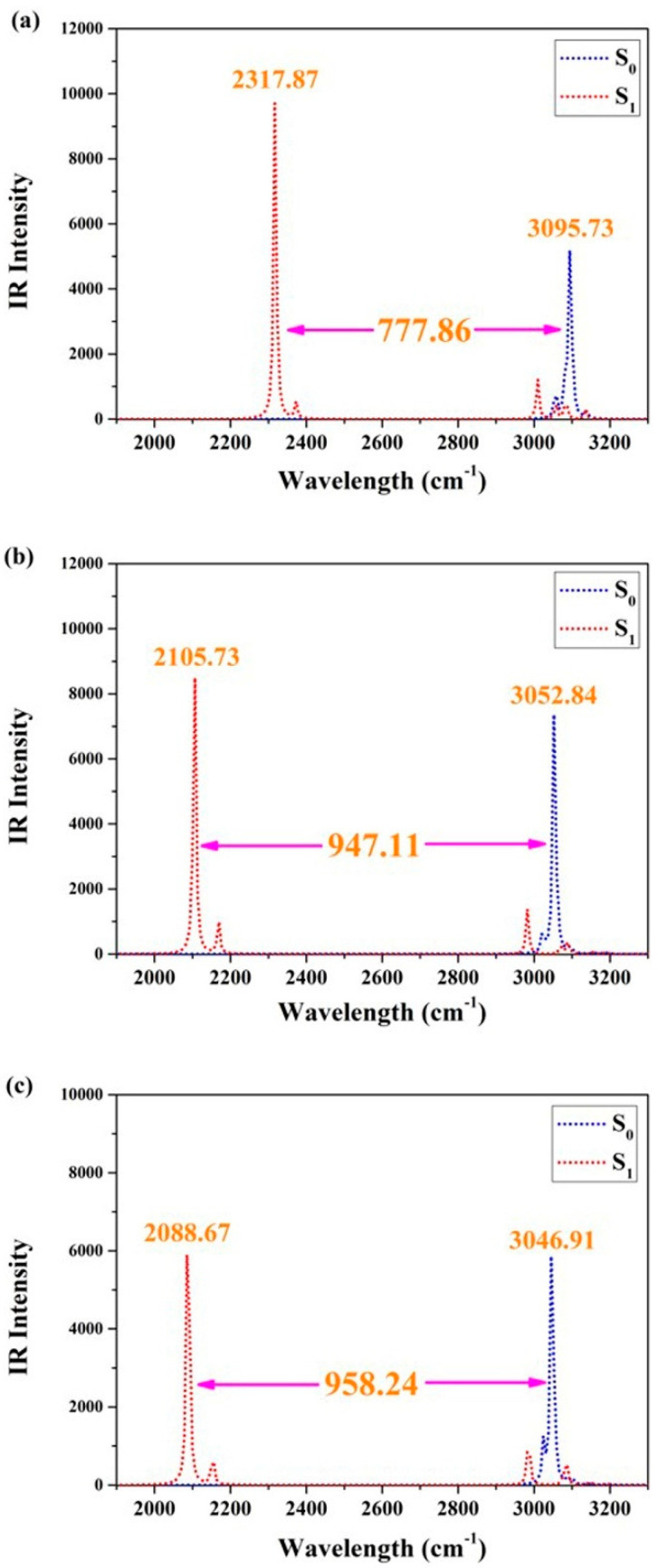
IR spectra related to synergetic O1–H2 and the O4–H5 stretching vibrational mode in the S_0_ and S_1_ states of BDYBD-O (**a**), BDYBD-S (**b**), and BDYBD-Se (**c**).

To further clarify the qualitative changes in hydrogen bonding effects after photoexcitation, we focused on investigating the vibrational behaviors of O1–H2 and O4–H5 using infrared (IR) vibrational spectra. Considering the practicality of IR analyses in determining bond strengths between atoms, it cannot be denied that this technique has become an essential tool for modern theoretical chemistry explorations [36,37,38,39,40]. Figure 2 presents the IR results of BDYBD-O, BDYBD-S, and BDYBD-Se. The stretching IR results of synergetic O1–H2 and O4–H5 vibration all exhibit a redshift in the S_1_ state, indicating that photoexcitation results in enhanced hydrogen bonding interactions for BDYBD-O, BDYBD-S, and BDYBD-Se [36,37,38,39,40]. It is noteworthy that the photo-induced redshifts of the IR spectral behaviors for BDYBD-O, BDYBD-S, and BDYBD-Se are different. To be specific, the redshifts of BDYBD-O, BDYBD-S, and BDYBD-Se are 777.86, 947.11, and 958.24 cm^−1^, respectively. This faintly reflects how the hydrogen bond interactions of the low-electronegativity BDYBD-Se compound become much stronger.

### 2.2. Hydrogen Bonding Strength

To provide an enlightening perspective on the quantitative evaluation of hydrogen bonding strength in relation to dual hydrogen bonds O1–H2···N3 and O4–H5···N6, we further carried out calculations for the core-valence bifurcation (CVB) index using the electron localization function (ELF) in the S_0_ and S_1_ states. As determined using Multiwfn v2.2 software [41], the simulated CVB indexes related to the dual hydrogen bonds for BDYBD-O, BDYBD-S, and BDYBD-Se are listed in Table S1 (ESI†). Because of the symmetry of the structures of BDYBD-O, BDYBD-S, and BDYBD-Se, the CVB indexes along the O1–H2···N3 and O4–H5···N6 hydrogen bonds should be the same. This empirical evidence suggests that CVB tends to exhibit a predominantly negative nature when exceptionally robust hydrogen bonds with covalent characteristics are present. However, in cases where the strength of hydrogen bonds is moderate, the CVB value approaches zero. A positive CVB result indicates weaker hydrogen bonding effects, whereas a negative CVB index suggests the formation of stronger hydrogen bonds [41]. From Table S1, it can be clearly seen that the S_0_-state CVB indexes with an order of hydrogen bond energy of O → S → Se change from moderate to strong strength [41]. For the S_1_ state, the simulated CVB indexes all changed and became more negative due to photoexcitation. One noteworthy aspect is that the CVB index progressively decreases with the change in O → S → Se order, indicating that the weakening of atomic electronegativity promotes the strengthening of excited-state hydrogen bonds in BDYBD derivatives.

In addition, we used the atom in molecule (AIM) method to analyze the electron density distribution of BDYBD-O, BDYBD-S, and BDYBD-Se [42]. The bond critical point (BCP) parameters between the acceptor and hydrogen atoms are shown in Table 2. It is clear that there are strong hydrogen bonding interactions between the S_0_ and S_1_ states of BDYBD-O, BDYBD-S, and BDYBD-Se. As is known, electron density (ρ(r)) is the key factor in determining the strength of chemical bonds. We noticed that S_1_-state ρ(r) values could be more negative than those of the S_0_ state, demonstrating the stronger hydrogen bonding effects in the S_1_ state due to photoexcitation. Moreover, in an ethanol solvent, we investigated both ρ(r) and hydrogen bonding energy (E_HB_) for BDYBD-O, BDYBD-S, and BDYBD-Se derivatives. The predicted E_HB_ can be calculated using the following formula: E_HB_ ≈ −223.08 × ρ(r) + 0.7423 [43]. Obviously, the higher values of ρ(r) and E_HB_ observed in BDYBD-Se fluorophore suggest that the hydrogen bonding is stronger in the Se element with the weakest atomic electronegativity, which helps enhance the ESIPT reactions of the BDYBD compounds.

### 2.3. Vertical Excitation Properties

The focus of this section is on the recombination of charges prior to and following light-induced excitation, as well as the alterations in the charge distributions among the key atoms involved in hydrogen bonding upon photoexcitation. In order to demonstrate the rationality of the calculation method, we initially conducted a comparison between the spectral behavior of the BDYBD-O molecular system using an ethanol solvent and the corresponding experimental values. Our computational absorption spectrum peak of BDYBD-O is located at 336.45 nm, which is consistent with our experimental result of 343.5 nm [34]. Most importantly, the fluorescence peak position calculated by us for the BDYBD-O-PT2 structure is located at 557.57 nm, which is also close to experimental value of 535 nm [44]. This fully reflects the rationality and correctness of the theoretical method we adopted in this study. In Table 3, the computational vertical excitation results for BDYBD-O, BDYBD-S, and BDYBD-Se are provided. Notably, their absorption peaks are at 336.45, 361.97, and 364.53 nm, respectively. Apparently, the absorption peaks exhibit a significant redshift upon excitation, accompanied by a decrease in atomic electronegativity from O to S to Se.

**Figure 3:**
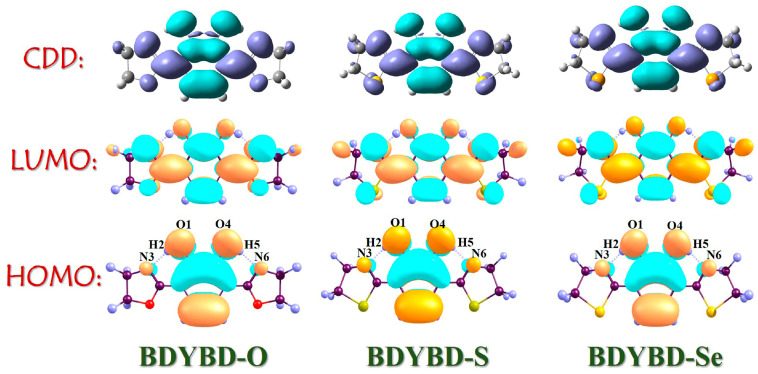
HOMO and LUMO orbitals and CDD maps of BDYBD-O, BDYBD-S, and BDYBD-Se.

To visually depict the charge distribution on the molecule pre- and post-photoexcitation [45,46,47,48,49,50], we employed frontier molecular orbitals (MOs) to investigate the dynamics of charge recombination for the BDYBD-O, BDYBD-S, and BDYBD-Se fluorophores. According to Kasha’s rule, we mainly focused on the effect of excitation of S_0_ to S_1_ on charge reorganization. As shown in Figure 3, the HOMO → LUMO transition belongs to the local excitation that presents the obvious π → π* transition behavior, which reveals the ππ*-type S_1_ state. The charge is distributed throughout the molecule; however, there are discernible variations in the sites associated with hydrogen bonding groups. After the HOMO → LUMO transition, it is evident that the charge distribution for the O1 and O4 atoms reduces, whereas that for the N3 and N6 atoms accrues. This observation indicates that following photoexcitation, the proton acceptors N3 and N6 exhibit enhanced hydrogen proton capture capabilities, whereas the proton donors O1 and O4 display an increased propensity to release hydrogen protons due to diminished electronegativity. Quantificationally, based on the Ros–Schuit partition [51], the contributions of O1 and O4 to HOMO sharply reduce from 29.03% (BDYBD-O), 28.71% (BDYBD-S), and 27.84% (BDYBD-Se) to 4.27%, 4.31%, and 4.29%, whereas those of N3 and N6 increase from 2.28% (BDYBD-O), 2.86% (BDYBD-S), and 3.49% (BDYBD-Se) to 20.09%, 24.26%, and 24.61%, respectively. Accordingly, we can deduce the driving force stemming from the photoexcitation impetus net charge densities shifting from the O1 and O4 to N3 and N6 atoms, which facilitates the ESIPT reaction behaviors for BDYBD-O, BDYBD-S, and BDYBD-Se.

The ESIPT phenomenon is primarily a result of the interaction between heavy and light atoms, leading to modifications. By utilizing advanced techniques such as Mulliken’s charge analysis and natural population analysis (NPA) charge methods, we can potentially gain a more profound understanding of the intricate mechanisms underlying this entire process. in Table S2 (ESI†), we list the simulated charge results related to the main atoms (O1, H2, N3, O4, H5, and N6) associated with the dual hydrogen bonds of BDYBD-O, BDYBD-S, and BDYBD-Se compounds. 

Our comparison between the Mulliken’s charge and NPA charge unveiled a consistent change trend in behavior, indicating that the atomic charge of the hydrogen bond acceptor becomes more negative upon photoexcitation, while the electronegativity of the hydrogen bond donor increases. This observation impeccably aligns with the conclusions derived from both the structural analysis and orbital analyses mentioned above.

### 2.4. Mechanism Exploration

**Figure 4:**
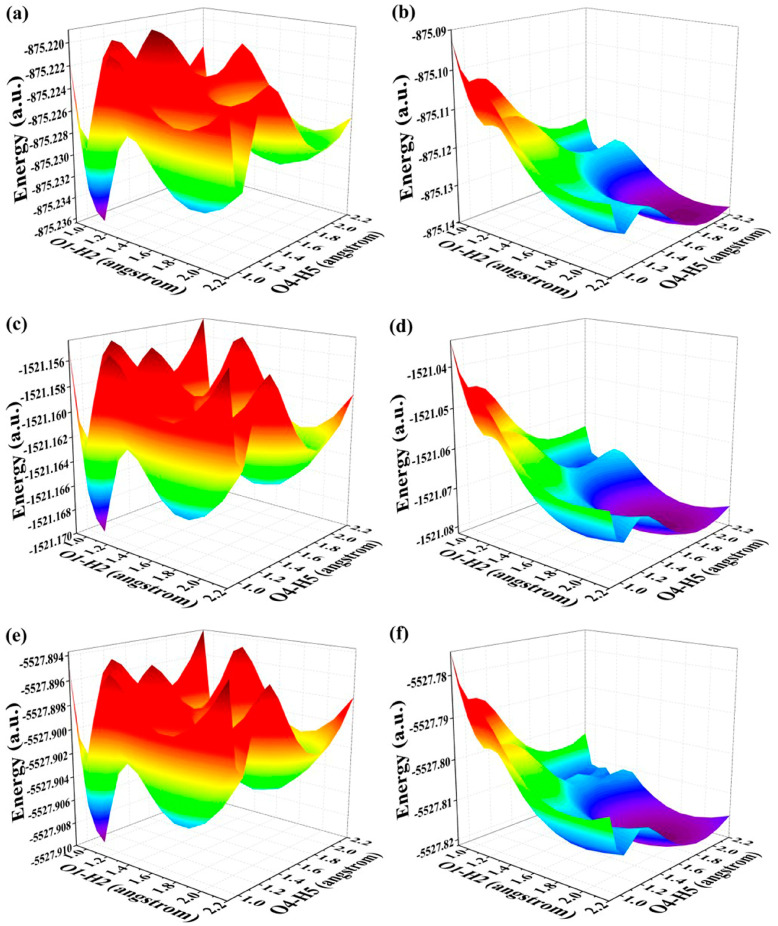
(**a**) S_0_-state PESs for BDYBD-O; (**b**) S_1_-state PESs for BDYBD-O; (**c**) S_0_-state PESs for BDYBD-S; (**d**) S_1_-state PESs for BDYBD-S; (**e**) S_0_-state PESs for BDYBD-Se; (**f**) S_1_-state PESs for BDYBD-Se.

In this section, our main focus is to elucidate the underlying mechanism governing the relevant ESDPT reaction. Given that BDYBD-O, BDYBD-S, and BDYBD-Se possess double hydrogen bonds O1–H2···N3 and O4–H5···N6, it is natural for us to direct our attention towards exploring the ESDPT process. In conjunction with these dual hydrogen bond pathways, we constructed potential energy surfaces (PESs) for both the S_0_ state and S_1_ state of these three compounds using a restrictive optimization approach [52,53,54]. Concretely speaking, optimizing the overall structures by adjusting the bond length of O1–H2 and O4–H5 within a range of 0.90 Å to 2.20 Å with an increment of 0.10 Å effectively elucidates the comprehensive kinetic process involved in this study for the BDYBD-O, BDYBD-S and BDYBD-Se fluorophores in both the S_0_ state and S_1_ state. The respective PESs could be formed by using the reaction coordinates of O1–H2 and O4–H5. The PESs for the S_0_-state and S_1_-state are illustrated in Figure 4. Interestingly, increasing the length of the O1–H2 and O4–H5 bonds consistently elevates the total potential energy, indicating that forward proton transfer reactions cannot occur for BDYBD-O, BDYBD-S, and BDYBD-Se in their respective S_0_ states.

**Figure 5:**
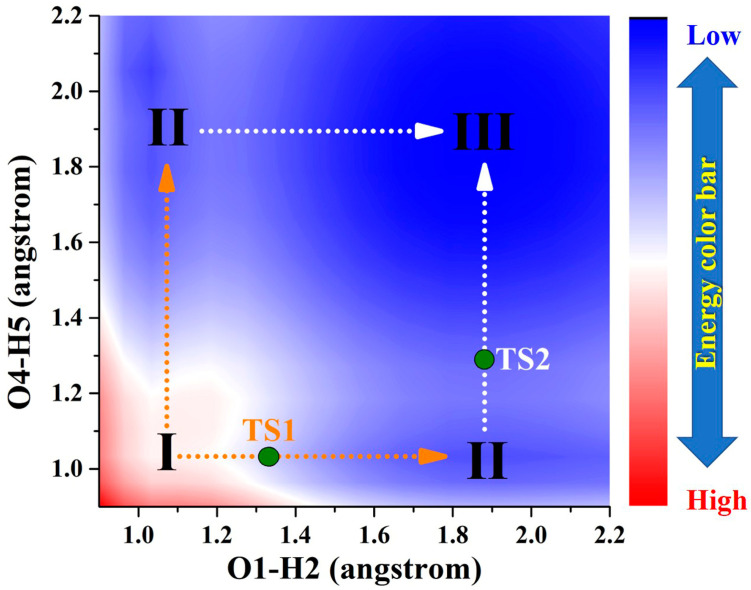
Projection plane of S_1_-state PESs for BDYBD derivatives. The TS1 and TS2 are marked along stepwise paths. I: S_1_-state BDYBD derivatives; II: BDYBD-PT1 derivatives; III; BDYBD-PT2 derivatives.

By comparison, as a result of photoexcitation, the three compounds exhibit remarkably distinct dynamical behaviors in the S_1_ state compared to that observed in the S_0_ state. In order to clearly describe the behavior in the excited state, a projection of the S_1_-state PESs of BDYBD is shown in Figure 5. Herein, we have labeled the stable molecular structures (I, II, and III) and the reaction process of forward ESIPT reaction. Herein, I stands for the S_1_-state optimized BDYBD-O, BDYBD-S, and BDYBD-Se structures; II refers to the S_1_-state optimized BDYBD-O-PT1, BDYBD-S-PT1, and BDYBD-Se-PT1 structures; and III represents the BDYBD-O-PT2, BDYBD-S-PT2, and BDYBD-Se-PT2 configurations. Transition state (TS) forms could be searched for via using the Berny optimization method [55]. The coordinates of all the TS configurations are shown in the Supplementary Materials. All the TS forms have been verified to own only one imaginary frequency, and the vibrational eigenvector points to the correct reaction direction. It is undeniable that symmetry in molecular structures can lead to symmetrical behavior in terms of potential energy surface structures. In the case of the S_1_ state, we can clearly see that the synergistic ESDPT behavior along the diagonal of the potential energy surface is untoward because the barrier of the synergistic ESDPT is larger than the barrier size of the single ESIPT along O1–H2···N3 or O4–H5···N6 for the BDYBD-O, BDYBD-S, and BDYBD-Se compounds. Therefore, we can rule out the possibility of collaborative ESDPT for sufficient reasons. In addition, it can be seen from the PES results that for the first-step ESIPT from BDYBD-O, BDYBD-S, and BDYBD-Se to BDYBD-O-PT1, BDYBD-S-PT1, and BDYBD-Se-PT1, the reaction energy barrier is small enough to result in ESIPT. This ESIPT reaction process can thus be easily carried out from an energy perspective. For the second ESIPT reaction from BDYBD-O-PT1, BDYBD-S-PT1, and BDYBD-Se-PT1 to BDYBD-O-PT2, BDYBD-S-PT2, and BDYBD-Se-PT2, we can see that the barrier becomes even lower than the first barrier of ESIPT, facilitating ESIPT behavior. The energies of the S_1_-state stepwise (I → II → III) and synergetic (I → III) ESDPT barrier sizes are listed in Table 4. To further confirm the correctness of the regulation of the ESDPT barrier by oxygen group elements, we recalculated the energy barrier sizes of stepwise and synergetic ESDPT by using D3-Cam-B3LYP/TZVP theory (Table S3). We can clearly see that the results calculated under Cam-B3LYP functional theory are consistent with the results calculated under B3LYP functional theory. In addition, considering the energy of molecularly stable structures, it can also be clearly seen that the energy values of the stable double-proton transfer isomers BDYBD-O-PT2, BDYBD-S-PT2, and BDYBD-Se-PT2 should be lower than those of BDYBD-O, BDYBD-S, BDYBD-Se, BDYBD-O-PT1, BDYBD-S-PT1, and BDYBD-Se-PT1. Thus, we can confirm the stepwise ESDPT mechanism for the chalcogen element-substituted BDYBD derivatives. The barrier values on the reaction path can be used to describe the effects of atomic electronegativities (O, S, Se) on the ESIPT behavior of BDYBD systems. We can clearly see in Table 4 that as the electronegativity of the atoms decreases (O → S → Se), the stepwise reaction energy barrier decreases accordingly. This indicates that the modulation of chalcogen atomic electronegativity exerts a regulatory influence on the ESDPT behavior of BDYBD derivatives, whereby lower atomic electronegativity is more conducive to the stepwise ESDPT reaction. 

## 3. Methods

All the computational results described in this work were technically derived from utilizing Gaussian 16 software [56]. DFT and TDDFT methods were employed to investigate the calculations of the S_0_ state and S_1_ states, respectively, based on B3LYP functional and TZVP basis sets [57,58,59,60]. To ensure the dispersion forces were considered throughout, in all simulations, we incorporated Grimme’s D3 version of dispersion to obtain the accurate results [61,62]. An ethanol solvent mentioned in a previous study was employed exclusively throughout all the calculations [34], which were carried out utilizing the IEFPCM approach [44,63,64]. All optimized geometries exhibited non-virtual frequencies, which ensured the stability of all the geometries. To gain insights into photo-induced charge redistribution, we carried out a vertical excitation simulation using the TDDFT method, starting from optimized S_0_-state configurations containing six low-lying absorption transitions. Using frontier molecular orbitals (Mos), the charge reorganization phenomenon could be clearly elucidated. To further explore the ESDPT mechanisms of the BDYBD derivatives associated with different chalcogen atomic substitutions, potential energy surfaces (PESs) were constructed by using a restrictive optimization approach and incorporating dual O1–H2···N3 and O4–H5···N6 through the implementation of a rigorous optimization method. Finally, the S_1_-state transition state (TS) forms were also searched for, along with stepwise ESDPT paths, using the Berny optimization method [55].

## 4. Conclusions

In summary, this work primarily focused on investigating the impact of chalcogen atomic electronegativity on double hydrogen bonding behaviors and ESDPT behaviors for three BDYBD derivatives (BDYBD-O, BDYBD-S, and BDYBD-Se). From a computational perspective, we initially evaluated the strengthening behavior of hydrogen bonds in the S_1_ state by emphasizing geometrical variations and IR spectral shifts. Our analyses of the CVB indexes and predictions of E_HB_ further demonstrated the adaptability of chalcogen electronegativity to the hydrogen bonding interactions. Upon gaining insights into the charge restructuring phenomenon deriving from photoexcitation, we confirmed the favorable tendency of charge reorganization around hydrogen bonding moieties that induces the ESDPT reaction behaviors among the BDYBD derivatives considered herein. Furthermore, we have successfully unraveled the intricate mechanism underlying the stepwise ESDPT reaction by constructing precise PESs for BDYBD-O, BDYBD-S, and BDYBD-Se fluorophores. Above all, we have proposed a pivotal approach for modulating ESDPT reactions in BDYBD derivatives based on the electronegativity of chalcogen atoms. We hope our research endeavors will pave an illustrious path towards pioneering applications for novel BDYBD derivatives.

## Figures and Tables

**Table 1:** Parameters of bond lengths (Å) and bond angles (Δ°) involved in O1–H2···N3 for BDYBD-O, BDYBD-S, and BDYBD-Se fluorophores in the S_0_ and S_1_ states.

	BDYBD-O		BDYBD-S		BDYBD-Se	
	S_0_	S_1_	S_0_	S_1_	S_0_	S_1_
O1–H2	0.999	1.042	1.001	1.056	1.001	1.057
H2···N3	1.709	1.576	1.687	1.528	1.684	1.526
Δ(O1H2N3)	147.51	151.17	147.82	152.06	147.86	152.03

**Table 2:** The electron density (ρ) based on the BCP parameters and predicted bonding energy values (kcal/mol) of O1–H2···N3 or O4–H5···N6 for BDYBD-O, BDYBD-S, and BDYBD-Se in the S_0_ and S_1_ states.

	S_0_		S_1_		∆ρ (S_1_–S_0_)	∆E (S_1_–S_0_)
	ρ	E_HB_	ρ	E_HB_	ρ	E_HB_
BDYBD-O	0.05211	−10.882	0.07187	−15.290	0.01976	4.408
BDYBD-S	0.05581	−11.708	0.08223	−17.602	0.02642	5.894
BDYBD-Se	0.05602	−11.755	0.08279	−17.726	0.02677	5.971

**Table 3:** Computational excitation energies (λ nm), oscillator strengths (*f*), transition compositions (CI), and related percentages for BDYBD-O, BDYBD-S, and BDYBD-Se.

	Transition	*λ*	*f*	Composition	CI (%)
BDYBD-O	S_0_ → S_1_	336.45	0.5885	H → L	97.24
	S_0_ → S_2_	279.76	0.0622	H-1 → L	96.61
	S_0_ → S_3_	251.44	0.0402	H-2 → L	97.48
BDYBD-S	S_0_ → S_1_	361.97	0.7153	H → L	97.68
	S_0_ → S_2_	311.36	0.0506	H-1 → L	92.07
	S_0_ → S_3_	290.62	0.0446	H-2 → L	98.28
BDYBD-Se	S_0_ → S_1_	364.53	0.5271	H → L	97.78
	S_0_ → S_2_	338.62	0.0527	H-1 → L	96.12
	S_0_ → S_3_	280.46	0.0356	H-2 → L	98.15

**Table 4:** The stepwise potential barriers (kcal/mol) in the S_1_ state along with I → III, I → II, and II → III paths for BDYBD-O, BDYBD-S, and BDYBD-Se.

	BDYBD-O	BDYBD-S	BDYBD-Se
I → III	3.0508	2.7893	2.2153
I → II	1.6354	1.2846	1.1421
II → III	2.7355	2.5954	2.244

## Data Availability

Data are available from the corresponding author upon request.

## Supplementary Materials

The following supporting information can be downloaded at: https://www.mdpi.com/article/10.3390/molecules29020461/s1, Table S1: The ELF parameters and CVB index involved in hydrogen bond O1–H2···N3 or O4–H5···N6 for BDYBD-O, BDYBD-S, and BDYBD-Se in the S_0_ and S_1_ states. Table S2: The calculated Mulliken charge and NPA charge of O1, H2, N3, O4, H5, and N6 atoms for BDYBD-O, BDYBD-S, and BDYBD-Se in both the S_0_ state and S_1_ state. Table S3: The stepwise potential barriers (kcal/mol) in the S_1_ state along with I → III, I → II, and II → III paths for BDYBD-O, BDYBD-S, and BDYBD-Se compounds using Cam-B3LYP functional. The coordinates of all searched TS forms in the S_1_ state.
