# Supplementary material for: Effects of Chalcogen Atoms on Excited-State Double-Proton Transfer Behavior for 3,6-bis(4,5-Dihydroxyoxazo-2-yl)benzene-1,2-diol Derivatives: A Computational Investigation
Source: Molecules. 2024 Jan 17;29(2):461. doi: 10.3390/molecules29020461 (PMC10820863; doi:10.3390/molecules29020461)
Supplement: Supplementary file 1 [file molecules-29-00461-s001.zip › molecules-2777449-supplementary.pdf]

Supporting Information

Effects of chalcogen atoms on excited state double proton transfer behavior for 3,6-bis(4,5-dihydroxyoxazo-2-yl)benzene-1,2-diol derivatives: A computational investigation

Dapeng Yang [1], Chang Liu [2], Meiyi Zhang [2], Jinfeng Zhao [2,3]*

[1.] College of Electronics and Engineering, North China University of Water Resources and Electric Power, Zhengzhou 450046, PR China.

[2.] College of Physical Science and Technology, Shenyang Normal University, Shenyang 110034, PR China.

[3.] Institute of Molecular Sciences and Engineering, Institute of Frontier and Interdisciplinary Science, Shandong University, Qingdao, China.

**To whom correspondence should be addressed*

E-mail Address: jfzhao1990112@synu.edu.cn; jfzhao1990112@163.com (J. Zhao)

Table S1. The ELF parameters and CVB index involved in hydrogen bond O1-H2···N3 or O4-H5···N6 for BDYBD-O, BDYBD-S and BDYBD-Se in S_0 and S_1 states. CVB index = ELF(C-V,D) - ELF(DH-A).

	BDYBD-O		BDYBD-S		BDYBD-Se	
States	S_0	S_1	S_0	S_1	S_0	S_1
ELF(C-V,D)	0.1026	0.1050	0.1035	0.1071	0.1036	0.1073
ELF(DH-A)	0.2183	0.3288	0.2369	0.3849	0.2373	0.3899
CVB index	-0.1157	-0.2238	-0.1334	-0.2778	-0.1337	-0.2826

Table S2. The calculated Mulliken charge and NPA charge of O1, H2, N3, O4, H5 and N6 atoms for BDYBD-O, BDYBD-S and BDYBD-Se in both S_0 and S_1 states.

		Mulliken charge		NPA charge	
	atoms	S_0	S_1	S_0	S_1
BDYBD-O	O1	-0.288	-0.285	-0.658	-0.656
	H2	0.295	0.290	0.504	0.497
	N3	-0.222	-0.229	-0.526	-0.528
	O4	-0.288	-0.285	-0.658	-0.656
	H5	0.295	0.290	0.504	0.497
	N6	-0.222	-0.229	-0.526	-0.528
BDYBD-S	O1	-0.290	-0.283	-0.655	-0.653
	H2	0.300	0.294	0.502	0.490
	N3	-0.176	-0.184	-0.482	-0.491
	O4	-0.290	-0.283	-0.655	-0.653
	H5	0.300	0.294	0.502	0.490
	N6	-0.176	-0.184	-0.482	-0.491
BDYBD-Se	O1	-0.291	-0.283	-0.654	-0.652
	H2	0.301	0.294	0.501	0.490
	N3	-0.185	-0.192	-0.484	-0.494
	O4	-0.291	-0.283	-0.654	-0.652
	H5	0.301	0.294	0.501	0.490
	N6	-0.185	-0.192	-0.484	-0.494

Table S3. The stepwise potential barriers (kcal/mol) in S_1 state along with I → III, I → II and II → III paths for BDYBD-O, BDYBD-S and BDYBD-Se compounds using Cam-B3LYP functional.

	BDYBD-O	BDYBD-S	BDYBD-Se
I → III	2.9548	2.6949	2.0541
I → II	1.6059	1.2473	1.1110
II → III	2.6872	2.4912	2.1017

The coordinates of the searched TS forms in S_1 state

BDYBD-O (TS1):

	X	Y	Z
C	0.72980900	-1.48773700	0.00363500
C	-0.67916800	-1.51523400	0.01179100
C	-1.43152000	-0.34823100	0.03227900
C	-0.72428600	0.92533600	-0.01494100
C	0.69253100	0.94191400	0.02085300
C	1.44582700	-0.29469500	-0.01298500
H	1.27225100	-2.42325900	-0.00419700
H	-1.18574200	-2.47117600	0.02268600
C	2.87363700	-0.23858400	0.00061900
O	3.59029600	-1.39822300	0.07079900
C	4.97425800	0.51447500	0.08907700
C	4.98136700	-1.01164100	-0.12841500
H	5.33826200	0.78850800	1.08513400
H	5.58940500	-1.56324500	0.58470300
C	-2.85101700	-0.30188700	0.03200600
O	-3.60966000	-1.42114700	-0.08997200
C	-4.92840300	0.54287200	-0.12083500
C	-4.99066000	-0.98096300	0.09535100
H	-5.20849200	0.82161100	-1.14309900
H	-5.60976900	-1.51123900	-0.62366400
N	3.55996400	0.86807100	-0.04649100
N	-3.51656800	0.83137700	0.12467800
H	5.58520000	1.03887700	-0.64643600
H	5.26389800	-1.28497700	-1.14685800
H	-5.57457200	1.08409500	0.56998400
H	-5.29134300	-1.24072600	1.11198800
O	1.31533200	2.11454400	0.03194400
H	2.32253400	1.92385900	0.01123900
O	-1.40159100	2.04530800	-0.06528000
H	-2.51466900	1.74393600	0.00067100

BDYBD-O (TS2):

	X	Y	Z
C	-0.71964400	-1.45552000	0.00978700
C	0.68040800	-1.50116100	0.00928700
C	1.42915100	-0.32724000	0.00568400
C	0.75127700	0.96030100	0.03231100
C	-0.70022500	1.02771500	-0.02533500

C	-1.42588400	-0.24535900	0.02202500
H	-1.27108200	-2.38637000	0.03123000
H	1.18389100	-2.45846500	0.00565700
C	-2.83656800	-0.24601800	0.04374100
O	-3.55491900	-1.38184000	-0.11217700
C	-5.00881900	0.46980200	-0.15468900
C	-4.96004200	-1.04251400	0.08057000
H	-5.20866200	0.71498400	-1.20330500
H	-5.54433100	-1.62446500	-0.62640200
C	2.85171500	-0.30953700	0.01254400
O	3.59733400	-1.42785000	-0.09891500
C	4.94646800	0.52554200	-0.10155100
C	4.98974200	-1.00649100	0.06965400
H	5.56158300	1.04037800	0.63556500
H	5.30552900	-1.30393300	1.07013400
N	-3.64353900	0.81978800	0.22607600
N	3.52602300	0.81033100	0.09343500
H	-5.74153700	0.96738800	0.47771200
H	-5.23388100	-1.30467200	1.10407600
H	5.26585500	0.83964300	-1.09967700
H	5.58516000	-1.52231500	-0.67884100
O	-1.30436200	2.13812500	-0.10034900
H	-3.19998500	1.73095800	0.10062500
O	1.45030900	2.05827400	0.04394000
H	2.62771100	1.70524700	0.06248900

BDYBD-S (TS1):

	X	Y	Z
C	0.68716200	-1.34318000	0.04732200
C	-0.72269100	-1.32434100	0.05205600
C	-1.45361800	-0.14389600	0.00505600
C	-0.69998200	1.09197600	0.00714800
C	0.72141600	1.08017900	-0.02994800
C	1.44274900	-0.18076800	0.01993500
H	1.18458100	-2.30357000	0.09444500
H	-1.24310100	-2.27262000	0.08727200
C	2.87366400	-0.14076800	0.01451300
C	4.93958000	0.91012300	-0.11090700
C	5.37329900	-0.50268300	0.30302800
H	5.46926400	1.66661900	0.46975500
H	5.53845400	-0.57694700	1.37707100
C	-2.88948600	-0.09502100	-0.00535700
C	-4.97454400	0.89126500	-0.11700300

C	-5.37665900	-0.52808000	0.30948600
H	-5.22791100	1.05741600	-1.17212000
H	-6.25599500	-0.89322800	-0.21589800
N	3.50731900	1.01270500	0.10710800
N	-3.53862600	1.03635200	0.05603300
H	5.16538500	1.08094700	-1.17194000
H	6.25589500	-0.84723300	-0.23038500
H	-5.50547600	1.64044000	0.47226900
H	-5.53506800	-0.59850300	1.38493200
O	1.37702000	2.21241200	-0.08999000
H	2.46786800	1.92634100	-0.01740000
O	-1.31630000	2.26284600	0.00175800
H	-2.33175900	2.05975000	0.01918400
S	3.93255300	-1.57718800	-0.14319200
S	-3.91843300	-1.57117000	-0.13838800

BDYBD-S (TS2):

	X	Y	Z
C	0.69029600	-1.33783100	0.11786700
C	-0.70983800	-1.30036400	0.12131600
C	-1.43505600	-0.10796600	0.03474800
C	-0.71496500	1.16293400	-0.05570800
C	0.74177800	1.10861600	0.00491200
C	1.43746900	-0.16623000	0.04134800
H	1.18680800	-2.29704800	0.18814900
H	-1.24070800	-2.23741400	0.23732400
C	2.86929300	-0.14656500	0.02426700
C	4.94251000	0.90378200	-0.16406100
C	5.37899800	-0.50303700	0.26346200
H	5.46511400	1.66904700	0.40986500
H	5.58063400	-0.55772000	1.33203700
C	-2.85981000	-0.11917400	0.05896900
C	-5.01379500	0.86845700	-0.04680700
C	-5.37609300	-0.58495100	0.24481800
H	-5.17358700	1.10210900	-1.10653700
H	-6.22813100	-0.93255200	-0.33305400
N	3.50766000	0.99601200	0.05789800
N	-3.60390700	0.98453500	0.29680400
H	5.15412900	1.07028100	-1.22656100
H	6.24041600	-0.86341200	-0.29300000
H	-5.60751100	1.55472100	0.55678100
H	-5.55121600	-0.75319300	1.30622000
O	1.41864700	2.21678000	-0.03819000

H	2.58318400	1.87371600	-0.00185300
O	-1.31062200	2.27453900	-0.17041600
H	-3.07829800	1.86280600	0.19870500
S	3.91973400	-1.58387600	-0.10781000
S	-3.87136000	-1.53810800	-0.26424800

BDYBD-Se (TS1):

	X	Y	Z
C	0.68558800	-1.00170100	0.09586100
C	-0.72394600	-0.98252700	0.10092500
C	-1.45690300	0.19490800	0.02469600
C	-0.70183700	1.43014800	0.00167300
C	0.71989700	1.41754300	-0.03642900
C	1.44319700	0.15816600	0.04165400
H	1.18109100	-1.96174900	0.16802300
H	-1.24269600	-1.93078600	0.15939100
C	2.87416600	0.19734500	0.03325400
C	4.93862400	1.29728000	-0.11818900
C	5.47432400	-0.05301600	0.37061600
H	5.42594200	2.11757700	0.41202700
H	5.61109500	-0.07429800	1.44982000
C	-2.89265400	0.24283900	0.01138800
C	-4.97851700	1.27624300	-0.12904600
C	-5.48250600	-0.08198700	0.37472500
H	-5.21775600	1.39293100	-1.19430700
H	-6.40028800	-0.39554900	-0.11646600
N	3.50482100	1.35150500	0.09734600
N	-3.53997900	1.37261600	0.04211700
H	5.15238100	1.41904000	-1.18878500
H	6.39424400	-0.34523500	-0.12929600
H	-5.47045000	2.08969800	0.40773100
H	-5.61348600	-0.09662000	1.45486200
O	1.37427000	2.54855300	-0.12023200
H	2.46443900	2.26201100	-0.04214300
O	-1.31689600	2.60120700	-0.02821300
H	-2.33145000	2.39782000	-0.00963400
Se	4.03832600	-1.34931800	-0.08769400
Se	-4.02546000	-1.34813000	-0.08250200

BDYBD-Se (TS2):

	X	Y	Z

C	-0.71251100	-0.95456100	0.23530600
C	0.68608700	-0.99072600	0.19874400
C	1.43269000	0.17709400	0.07042200
C	0.73179900	1.44459100	-0.03642900
C	-0.72515800	1.50274200	0.00899100
C	-1.44554000	0.22916400	0.10092000
H	-1.23771200	-1.88886700	0.39433000
H	1.18489100	-1.94470900	0.31376200
C	-2.86880400	0.21054400	0.12534500
C	-5.02804500	1.23922200	-0.02490300
C	-5.49720500	-0.17036900	0.31460200
H	-5.16316700	1.43958400	-1.09525300
H	-6.37548100	-0.46830800	-0.25098800
C	2.86308100	0.19607600	0.02102600
C	4.93096200	1.28620900	-0.26444700
C	5.47618200	-0.03112300	0.29428700
H	5.41280800	2.13899600	0.21560400
H	5.65160600	0.01883400	1.36667800
N	-3.61845700	1.31396200	0.32925600
N	3.49642200	1.33831800	-0.03567700
H	-5.58838000	1.98506400	0.54001000
H	-5.67027400	-0.30125300	1.38035900
H	5.12264600	1.35333500	-1.34194800
H	6.37651900	-0.36090500	-0.21715700
O	-1.32319700	2.61649400	-0.05429500
H	-3.08330000	2.18987000	0.25995500
O	1.40206700	2.54800600	-0.18664700
H	2.56554400	2.20789000	-0.14619600
Se	-3.96479000	-1.32658100	-0.20853200
Se	4.02262400	-1.35238000	-0.02116900
